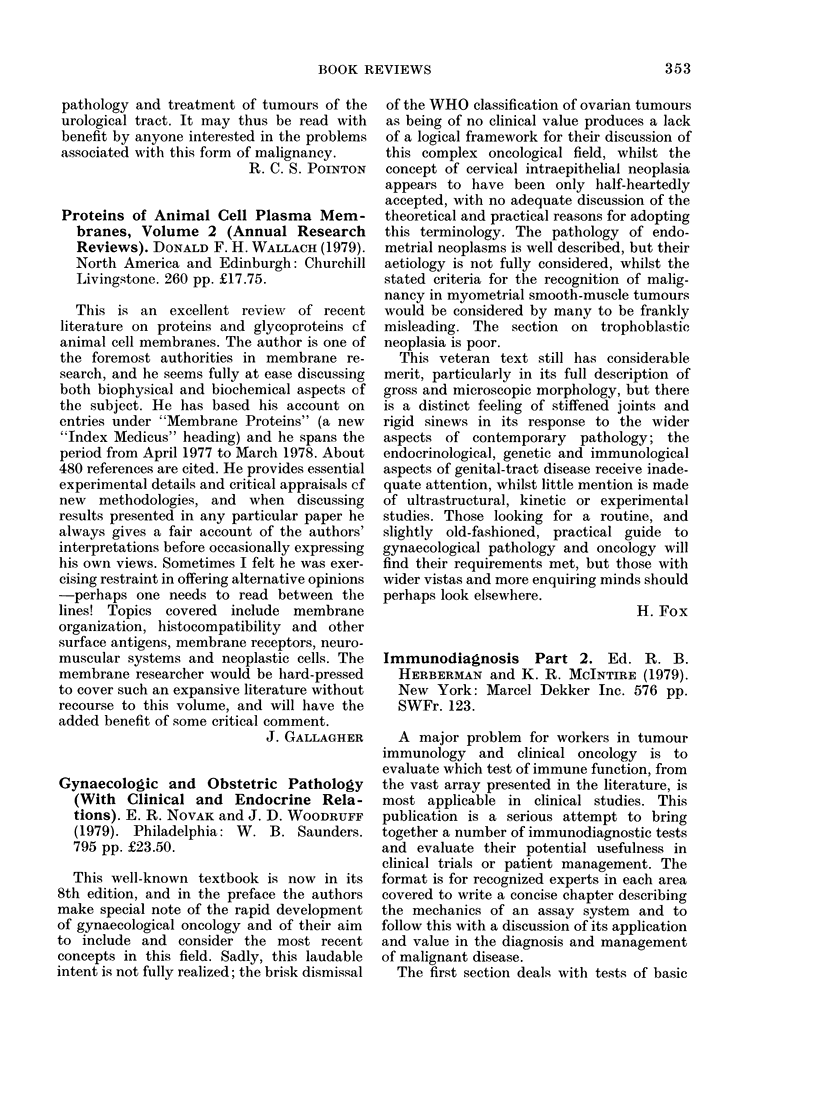# Proteins of Animal Cell Plasma Membranes, Volume 2 (Annual Research Reviews)

**Published:** 1980-08

**Authors:** J. Gallagher


**Proteins of Animal Cell Plasma Membranes, Volume 2 (Annual Research Reviews).** Donald F. H. Wallach (1979). North America and Edinburgh: Churchill Livingstone. 260 pp. £17.75.

This is an excellent review of recent literature on proteins and glycoproteins of animal cell membranes. The author is one of the foremost authorities in membrane research, and he seems fully at ease discussing both biophysical and biochemical aspects of the subject. He has based his account on entries under "Membrane Proteins" (a new "Index Medicus" heading) and he spans the period from April 1977 to March 1978. About 480 references are cited. He provides essential experimental details and critical appraisals of new methodologies, and when discussing results presented in any particular paper he always gives a fair account of the authors' interpretations before occasionally expressing his own views. Sometimes I felt he was exercising restraint in offering alternative opinions —perhaps one needs to read between the lines! Topics covered include membrane organization, histocompatibility and other surface antigens, membrane receptors, neuromuscular systems and neoplastic cells. The membrane researcher would be hard-pressed to cover such an expansive literature without recourse to this volume, and will have the added benefit of some critical comment.

J. Gallagher